# Compliance with tobacco advertising and promotion laws at points-of-sale in Ethiopia: an observational study in 10 cities

**DOI:** 10.1186/s12889-024-19478-7

**Published:** 2024-07-22

**Authors:** Wakgari Deressa, Selamawit Hirpa, Terefe Gelibo Argefa, Awgichew Kifle, Selam Abraham Kassa, Rachel Kitonyo-Devotsu, Winnie Awuor, Noreen Dadirai Mdege

**Affiliations:** 1 Department of Preventive Medicine, School of Public Health, College of Health Sciences, Addis Ababa University, Addis Ababa, Ethiopia; 2 Development Gateway: an IREX Venture, Washington, DC USA; 3 Mailman School of Public Health, ICAP at Columbia University, Addis Ababa, Ethiopia; 4 Department of Health Sciences, University of York, York, YO10 5DD UK; 5 Centre for Research in Health and Development, York, UK

**Keywords:** Tobacco, Advertising, Promotion, TAPS, Smoking, Ethiopia

## Abstract

**Background:**

Ethiopia enacted a comprehensive tobacco control law in 2019, which bans tobacco advertising and promotion activities. However, compliance with these laws at points-of-sale (PoS) has not been studied, resulting in a lack of research evidence on how the regulations are implemented. The purpose of the study was to assess compliance with tobacco advertising and promotion laws at PoS in 10 cities in Ethiopia.

**Methods:**

Multi-stage cluster sampling was used to select 1468 PoS (supermarkets, minimarkets, merchandise stores, regular shops, permanent kiosks, khat shops, street vendors, and food and drink wholesalers). Data were collected using standardized observational checklists. Tobacco advertising and promotion indicators were used to compute indoor and outdoor compliance. Poisson regression models with log link function and robust variance were used to assess factors associated with open display of cigarette packages and indoor non-compliance.

**Results:**

The average indoor compliance rate was 92.9% (95% CI:92.3–93.5). Supermarkets had the highest compliance (99.7%), while permanent kiosks showed the lowest compliance (89.8%). The highest average indoor compliance was observed at PoS in Addis Ababa (98.0%). About 60% of PoS were fully compliant in indoors. Indoor open display of cigarette packages was prevalent (32.5%, 95% CI:30.0-35.1). The average outdoor compliance was 99.6% (95% CI:99.5–99.7). Outdoor full compliance was 96.5%. Open display of cigarettes was significantly higher in permanent kiosks (adjusted prevalence ratio (adjPR) 6.73; 95% CI: 3.96–11.42), regular shops (adjPR 5.16; 95% CI: 3.05–8.75), and khat shops (adjPR 2.06; 95% CI: 1.11–3.83), while indoor non-compliance was significantly higher in these same types of PoS.

**Conclusions:**

While outdoor compliance rates were relatively high, the lower indoor compliance rates particularly due to the high prevalence of open cigarette package displays indicates a major area for improvement in enforcing anti-tobacco advertising and promotion laws.

**Supplementary Information:**

The online version contains supplementary material available at 10.1186/s12889-024-19478-7.

## Background

The tobacco industry employs a variety of strategies, including the use of retail outlets to promote and sell tobacco products [1]. As a result of tobacco advertising and promotion bans in many countries [2], points-of-sale (PoS) have been utilized by the tobacco industry as the first-line of advertising and promotion, for example, through open display of tobacco products [1, 3, 4]. Article 13 of the World Health Organization (WHO) Framework Convention on Tobacco Control (FCTC) [5] states that openly displaying tobacco products at the PoS constitutes advertising and promotion of tobacco use.

Exposing adolescents and youth to tobacco advertising, promotions, and product displays at the PoS can increase intentions to smoke, susceptibility to smoking, or smoking initiation [6–10]. Studies have shown that tobacco product advertising and promotion are more common near schools [11], and youth smoking rates have been found to be higher in schools located in areas with a higher density of tobacco outlets and cigarette advertising [12]. Moreover, studies have shown that exposure to tobacco outlets near home environments is associated with adolescents’ smoking behavior [13].

About 5% of adults in Ethiopia use tobacco products [14–16]. In 2019, Ethiopia adopted a new comprehensive tobacco control law (Proclamation No. 1112/2019) that prohibits both direct and indirect tobacco advertising, promotion, and sponsorship (TAPS) including the open display of tobacco products at PoS [17]. According to the Proclamation, tobacco products at the PoS should be placed under or behind the counter or in similar arrangements so that customers cannot directly see or grasp the product. It is also forbidden to display any kind of advertisement at PoS, and that includes the trademark, logo, or emblem of the tobacco industry, or banners, posters, stickers, plastic bags, watches, umbrellas, or any other furniture or object that advertises tobacco industry or promote tobacco product.

Banning advertising and the open display of tobacco products at the PoS can lower exposure to cigarette and tobacco use prevalence [10, 12, 18]. It can also reduce impulse purchases of tobacco products by smokers [19, 20]. However, enforcement of tobacco advertising and promotion bans at PoS is challenging for many countries, particularly low- and middle-income countries (LMICs) [3, 21, 22]. For example, studies in Lebanon [23] and India [24, 25] have shown low compliance with tobacco advertising and promotion bans at PoS, while studies from Europe have shown high levels of compliance [18, 26, 27].

Compliance monitoring is a key component of tobacco control policy implementation [5]. Unfortunately, no studies have evaluated compliance with tobacco advertising and promotion bans at PoS in Ethiopia [28]. The purpose of this study was to address this evidence gap by assessing the level of compliance with tobacco advertising and promotion laws at PoS in 10 major cities of Ethiopia. In this study, we defined PoS as any retail establishment where cigarettes and other tobacco products can be sold directly to individuals. Tobacco advertising constitutes any form of commercial communication, recommendation, or action by the tobacco industry or its business partners with the intention, effect or likely effect of promoting a tobacco product or tobacco use, whether directly or indirectly [5].

## Methods

### Study design and settings

A cross-sectional observational study of compliance was conducted in December 2022 in 10 major regional and chartered cities in Ethiopia: Addis Ababa, Adama, Assosa, Bahir Dar, Dire Dawa, Gambella, Harar, Hawassa, Jigjiga, and Semera-Logia (where Semera and Logia are two separate nearby cities merged as one study area). These cities have high population density and smoking prevalence [16], varying levels of tobacco control intervention efforts, and diverse cultural, economic, and social characteristics. Due to security problems, this study was not carried out in Tigray Regional State.

### Study population and sample size determination

The study population consisted of PoS that sell tobacco products, including regular shops, supermarkets, minimarkets, merchandise stores, permanent kiosks, street vendors, khat shops, and food and drink wholesalers located in the selected cities. We used a single proportion sample size formula to determine the minimum sample size required for the study. In the absence of similar studies in Africa, we assumed a 50% compliance with tobacco advertising and promotion laws at PoS. Assuming a 95% confidence level with 4% margin of error, and a design effect of two, a minimum of 1300 PoS were required for the study. The sample size was proportionally allocated among the 10 cities considering their estimated population size.

### Sampling procedures

Administratively, each city is organized into sub-cities and/or districts (or woreda). The lowest administrative unit considered for this study was the woreda (or kebele in certain cities). The woredas (or kebeles) were chosen from a list of woredas/kebeles in each city/sub-city (S1 File). In each kebele/woreda/sub-city, 4–6 neighborhoods consisting of various streets with the highest density of PoS were selected in consultation with the woreda or kebele offices [29]. In this study, neighborhood was defined as a business or residential area with streets as their boundaries. Next, all streets, typically 4–6 streets in each selected neighborhood, were identified and mapped in more detail by directly observing both sides of each street and listing all retail establishments [29]. This procedure was complemented with random walk methods for those streets where mapping and listing were challenging.

### Data collection tools and procedures

This study used standardized observational checklists to guide the direct observation of compliance with tobacco advertising and promotion laws. The observation checklists were adapted from the compliance assessment guidance “How-to-Guide for Conducting Compliance Studies” for TAPS bans [29]. The adaptations were to ensure that the checklists adequately covered the provisions of the Ethiopian Tobacco Control Proclamation (1112/2019) [17]. The checklists were initially developed in English, translated into Amharic, and then back into English.

The outdoor observation checklist asked whether there were any outdoor advertisements, what kinds of outdoor advertisements (such as posters, freestanding umbrella or billboard, wall paints/decorations) were present, and whether they were clearly visible from a point of regular pedestrian or vehicle traffic. The indoor observation checklist included questions about the types of visible tobacco advertisements (such as banners, posters, stickers, or any other material used for tobacco advertisements and promotion), whether or not tobacco products can be seen directly from the entrance, main counter, or any other area of the PoS, as well as questions about open displays of tobacco products, types of advertisements and promotions, and tobacco product placement.

Data were collected by 24 data collectors recruited from the universities located in the cities in which the study was being conducted. Data collection at each PoS was conducted by two data collectors. The data collectors were supervised by 10 field supervisors. A three-day training was held for data collectors and field supervisors. The observation checklists were pre-tested, modified, and adjusted as necessary. Tobacco control experts from the Ethiopian Food and Drug Authority (EFDA) also reviewed the observation checklists (S2 File). In this study, compliance was assessed by calculating the average compliance rate across multiple compliance indicators, evaluating the extent to which the PoS was complying with tobacco control laws under the 2019 Ethiopian Tobacco Control Law (Proclamation No.1112/2019). S3 File presents further operational definitions used in this study.

Data were collected in two stages: (1) covert observation of tobacco advertisements and promotional items located at the outdoor space; and (2) covert observation of tobacco advertisements and promotions inside the establishment. The data collectors spent a few minutes outside the establishment observing the outdoor tobacco advertising. The indoor observations were made from the entrance, or main counter, or any other part of the establishment (for example, walking through the aisles in supermarkets and mini-markets). The data were entered into the smartphone/tablet using Open Data Kit (ODK). Because owners of the observed establishment were not aware of the study, the reliability and validity of the collected data were higher than if they had been informed about the study beforehand [30]. Data were collected between 09:00–18:00 h from December 05 to 28, 2022.

#### Indoor compliance

A composite indoor compliance indicator was created from 23 specific compliance indicators, which were coded as 0 = no and 1 = yes (S4 File). The code ‘0 = no’ indicated non-compliance, whereas the code ‘1 = yes’ indicated compliance.

#### Outdoor compliance

A composite outdoor compliance indicator was also created using 10 individual indicators of compliance with tobacco advertising and promotion laws, which comprised (1) No tobacco advertising poster, (2) No tobacco advertising plastic bag, (3) No tobacco advertising freestanding umbrella, (4) No wall paintings/decoration with tobacco advertisement, (5) No cigarette carton boxes, (6) No outdoor tobacco advertisements with culturally specific references (such as special images, symbols, or colors, etc.), (7) No e-cigarette outdoor advertisement, (8) No tobacco advertising freestanding billboard, (9) No public TV screen showing tobacco advertisements, and (10) No transit vehicles with tobacco advertisements. Each of these specific indicators was coded as 0 = no and 1 = yes. The code ‘0 = no’ indicated non-compliance, whereas the code ‘1 = yes’ showed compliance.

### Data analysis

Descriptive analysis and tabulations were used to summarize and present the data. Compliance was calculated for each specific indicator. Average indoor and outdoor compliances with tobacco advertising and promotion laws across all types of PoS and cities were computed. We summed up the indicator-specific indoor compliance estimates and divided the sum by the number of indoor indicators in order to obtain the average indoor compliance. Similarly, the sum of the indicator-specific outdoor compliance estimates was divided by the number of outdoor indicators in order to obtain average outdoor compliance.

Additionally, a PoS was rated as ‘fully compliant’ for indoor compliance if all of the 23 criteria were met, and ‘non-compliant’ if any one of the criteria were not full-filled. The indoor compliance was further rated as ‘good compliant’ when 20 to 22 indicators were met, ‘moderately compliant’ when 15 to 19 indicators were met, and ‘poorly compliant’ when 14 or less indicators were met. Similarly, a PoS was rated as ‘fully compliant’ for outdoor compliance if it scored ‘yes’ for all the 10 outdoor compliance indicators, otherwise it was considered to be non-compliant. Further, the outdoor compliance was classified into ‘good compliance’ when nine indicators were met, and ‘moderately compliant’ when eight indicators were met.

To assess the relationship between indoor non-compliance or open display of cigarettes in the indoors (dependent variables), and independent variables (types of establishments and cities), adjusted prevalence ratios (adjPR) with 95% confidence intervals (CIs) were calculated using Poisson regression models with log link function and robust variance as recommended for frequent outcomes [33]. We tested for multicollinearity between the types of PoS and cities using the Variance Inflation Factor (VIF), and found no evidence of collinearity (VIF < 2). We estimated standard errors while accounting for the complex sample design, including adjusting for stratification by city and clustering of PoS at kebele/village level. *P* < 0.05 was selected for the significance tests. We performed all analysis using SPSS version 26 (IBM SPSS Statistics for Windows, Armonk, NY, USA) and Stata version 14 (StataCorp LP, College Station, TX, USA).

## Results

### Characteristics of the points-of-sale

A total of 1468 PoS were observed across the 10 cities. Regular shops accounted for more than half (51.2%) of the total, followed by khat shops (11.2%), street vendors (9.9%), permanent kiosks (9.2%), minimarkets (6.9%), merchandise stores (6.1%), supermarkets (3.3%), and food and drink wholesalers (2.3%) (Table 1). Addis Ababa accounted for 17.5% (*n* = 257) of the total PoS, with the number of PoS in the remaining cities ranging from 110 (7.5%) each in Assosa and Dire Dawa to 162 (11.0%) in Adama.

**Table 1:** Types of the points-of-sale by city

City	Points-of-sale, *n* (%)								Total, *n* (%)
	Regular shop	Khat shop	Street vendor	Permanent kiosk	Minimarket	Merchandise store	Supermarket	Food & drink wholesaler	
**Addis Ababa**	109 (14.5)	60 (36.6)	26 (17.9)	9 (6.7)	9 (8.9)	33 (37.1)	5 (10.4)	6 (17.6)	257 (17.5)
**Adama**	48 (6.4)	33 (20.1)	17 (11.7)	31 (23.0)	17 (16.8)	0.0	16 (33.3)	0.0	162 (11.0)
**Hawassa**	83 (11.0)	11 (6.7)	16 (11.0)	8 (5.9)	9 (8.9)	15 (16.9)	9 (18.8)	2 (5.9)	153 (10.4)
**Bahir Dar**	86 (11.4)	24 (14.6)	16 (11.0)	0.0	21 (20.8)	0.0	3 (6.3)	0.0	150 (10.2)
**Harar**	73 (9.7)	0.0	14 (9.7)	2 (1.5)	20 (19.8)	9 (10.1)	4 (8.3)	15 (44.1)	137 (9.3)
**Gambella**	72 (9.6)	24 (14.6)	12 (8.3)	0.0	5 (5.0)	20 (22.5)	2 (4.2)	1 (2.9)	136 (9.3)
**Semera-Logia**	74 (9.8)	1 (0.6)	14 (9.7)	26 (19.3)	5 (5.0)	4 (4.5)	0.0	8 (23.5)	132 (9.0)
**Jigjiga**	82 (11.4)	1 (0.6)	10 (6.9)	12 (8.9)	7 (6.9)	4 (4.5)	3 (6.3)	2 (5.9)	121 (8.2)
**Dire Dawa**	43 (5.7)	0.0	10 (6.9)	47 (34.8)	4 (4.0)	3 (3.4)	3 (6.3)	0.0	110 (7.5)
**Assosa**	82 (10.9)	10 (6.1)	10 (6.9)	0.0	4 (4.0)	1 (1.1)	3 (6.3)	0.0	110 (7.5)
**Total**,** n (%)**	**752 (51.2)**	**164 (11.2)**	**145 (9.9)**	**135 (9.2)**	**101 (6.9)**	**89 (6.1)**	**48 (3.3)**	**34 (2.3)**	**1**,**468 (100)**

### Indoor compliance with tobacco advertising and promotion laws

The most common types of indoor tobacco advertising and promotion were open display of cigarette packages (32.5%), visible cigarettes display on shelves (28.9%), display of cigarette package with inaccurate or misleading information that can attract customers (27.8%), cigarettes display near cashiers (20.6%), cigarettes display among products for children (14.5%), and cigarettes display above the counter (14.1%) (Table 2). Supermarkets (2.1%) and minimarkets (11.9%) had the lowest proportions of PoS with open cigarette displays.

**Table 2:** Indoor compliance indicators of tobacco advertising and promotion laws by points-of-sale

No.	Indoor compliance indicators (*n* = 1323)	Points-of-sale, *n* (%)							
		Supermarket, *n* = 48	Minimarket, *n* = 101	Merchandise store, *n* = 89	Food & drink wholesaler, *n* = 34	Regular shop, *n* = 752	Permanent kiosk, *n* = 135	Khat shop, *n* = 164	Overall compliance
1	No tobacco product advertisements clearly visible from the entrance, main counter, or any other part of the PoS	48 (100.0)	100 (99.0)	84 (94.4)	31 (91.2)	709 (94.3)	124 (91.9)	161 (98.2)	**95.0**
2	No poster with tobacco product advertisement	48 (100.0)	101 (100.0)	89 (100.0)	34 (100.0)	736 (97.9)	133 (98.5)	164 (100.0)	**98.6**
3	No price sticker of tobacco product	48 (100.0)	101 (100.0)	85 (95.5)	34 (100.0)	745 (99.1)	131 (97.0)	163 (99.4)	**98.8**
4	No video screen with tobacco product advertisements	48 (100.0)	101 (100.0)	89 (100.0)	34 (100.0)	752 (100.0)	135 (100.0)	164 (100.0)	**100.0**
5	No furniture/objects with tobacco product advertisements	48 (100.0)	101 (100.0)	89 (100.0)	34 (100.0)	752 (100.0)	135 (100.0)	164 (100.0)	**100.0**
6	No plastic bag with tobacco product advertisement	48 (100.0)	100 (99.0)	88 (98.9)	33 (97.1)	722 (96.0)	130 (96.3)	164 (100.0)	**97.1**
7	No uniform clothing with tobacco product advertisements	48 (100.0)	101 (100.0)	89 (100.0)	34 (100.0)	752 (100.0)	135 (100.0)	163 (99.4)	**99.9**
8	No watches with tobacco product advertisements	48 (100.0)	101 (100.0)	89 (100.0)	34 (100.0)	752 (100.0)	135 (100.0)	164 (100.0)	**100.0**
9	No umbrellas with tobacco product advertisements	48 (100.0)	101 (100.0)	89 (100.0)	34 (100.0)	752 (100.0)	135 (100.0)	164 (100.0)	**100.0**
10	No cigarette package with inaccurate or misleading information that can attract customers	48 (100.0)	93 (92.1)	57 (64.0)	26 (76.5)	501 (92.1)	81 (60.0)	149 (90.9)	**72.2**
11	No price stickers that obscure health warnings on cigarette packages	48 (100.0)	101 (100.0)	82 (92.1)	34 (100.0)	715 (95.1)	122 (90.4)	161 (98.2)	**95.5**
12	No gifts with purchase of cigarette, special or limited time offer	48 (100.0)	101 (100.0)	87 (97.8)	34 (100.0)	748 (99.5)	134 (99.3)	164 (100.0)	**99.5**
13	No multi-pack discount for tobacco product	48 (100.0)	101 (100.0)	89 (100.0)	34 (100.0)	748 (99.5)	134 (99.3)	164 (100.0)	**99.6**
14	No tobacco advertisements with culturally specific references (*such as special images*,* symbols*,* or colors*)	48 (100.0)	101 (100.0)	89 (100.0)	34 (100.0)	752 (100.0)	135 (100.0)	164 (100.0)	**100.0**
15	No advertisements of e-cigarettes	48 (100.0)	101 (100.0)	89 (100.0)	34 (100.0)	752 (100.0)	135 (100.0)	164 (100.0)	**100.0**
16	No tobacco product clearly visible from the entrance/main counter or any other part of the PoS	47 (97.9)	89 (88.1)	65 (73.0)	27 (79.4)	461 (61.3)	66 (48.9)	138 (84.1)	**67.5**
17	No cigarette packages displayed near a cashier	48 (100.0)	100 (99.0)	72 (80.9)	31 (91.2)	561 (74.6)	89 (65.9)	149 (90.9)	**79.4**
18	No cigarette packages displayed above the counter	48 (100.0)	100 (99.0)	76 (85.4)	32 (94.1)	616 (81.9)	111 (82.2)	153 (93.3)	**85.9**
19	No cigarette packages displayed on shelves	47 (97.9)	89 (88.1)	66 (74.2)	27 (79.4)	490 (65.2)	73 (54.1)	149 (90.9)	**71.1**
20	No cigarettes displayed in any power wall	48 (100.0)	101 (100.0)	85 (95.5)	33 (97.1)	738 (98.1)	132 (97.8)	164 (100.0)	**98.3**
21	No cigarettes displayed near products for children	47 (97.9)	95 (94.1)	78 (87.6)	32 (94.1)	601 (79.9)	117 (86.7)	161 (98.2)	**85.5**
22	No cigarettes placed with candles	48 (100.0)	101 (100.0)	86 (96.6)	33 (97.1)	689 (91.6)	135 (100.0)	163 (99.4)	**94.9**
23	No cigarettes placed with attractive lights	48 (100.0)	101 (100.0)	88 (98.9)	33 (97.1)	733 (97.5)	130 (96.3)	164 (100.0)	**98.0**
	**Average compliance (%)**	**99.7**	**98.2**	**92.8**	**95.4**	**91.2**	**89.8**	**97.5**	**92.9**

According to the composite indicator constructed from 23 specific indoor compliance indicators, the average compliance was 92.9% (95% CI:92.3–93.5) (Table 2). The overall compliance across the indoor indicators ranged from 67.5% for ‘no tobacco product clearly visible from the entrance/main counter or any other part of the PoS’ to 100% for ‘no culturally specific tobacco advertisements’, ‘no tobacco promotion on video displays’, and ‘no tobacco advertising’ using furniture, watches, and umbrellas. Supermarkets (99.7%) and minimarkets (98.2%) had the highest level of average compliance, while permanent kiosks (89.8%) had the lowest average indoor compliance.

City-level compliance for each indoor indicator is shown in Table 3. Addis Ababa had the highest average indoor compliance (98.0%), followed by Jigjiga (96.4%), and Dire Dawa (95.6%), while Semera-Logia had the lowest average indoor compliance (80.5%).

**Table 3:** Indoor compliance indicators of tobacco advertising and promotion laws by city

No.	Indoor compliance indicators (*n* = 1323)	City, *n* (%)									
		Addis Ababa, *n* = 231	Adama, *n* = 145	Bahir Dar, *n* = 134	Hawassa, *n* = 137	Jigjiga, *n* = 111	Semera- Logia, *n* = 118	Dire Dawa, *n* = 100	Harar, *n* = 123	Assosa, *n* = 100	Gambella, *n* = 124
1	No tobacco product advertisements clearly visible from the entrance, main counter, or any other part of the PoS	227 (98.3)	140 (96.6)	132 (98.5)	133 (97.1)	107 (96.4)	84 (71.2)	98 (98.0)	112 (91.1)	100 (100.0)	124 (100.0)
2	No poster with tobacco product advertisement	231 (100.0)	144 (99.3)	134 (100.0)	136 (99.3)	111 (100.0)	102 (86.4)	100 (100.0)	123 (100.0)	100 (100.0)	124 (100.0)
3	No price sticker of tobacco product	231 (100.0)	145 (100.0)	134 (100.0)	134 (97.8)	111 (100.0)	105 (89.0)	100 (100.0).	123 (100.0)	100 (100.0)	124 (100.0)
4	No video screen with tobacco product advertisements	231 (100.0)	145 (100.0)	134 (100.0)	137 (100.0)	111 (100.0)	118 (100.0)	100 (100.0)	123 (100.0)	100 (100.0)	124 (100.0)
5	No furniture/objects with tobacco product advertisements	231 (100.0)	145 (100.0)	134 (100.0)	137 (100.0)	111 (100.0)	118 (100.0)	100 (100.0)	123 (100.0)	100 (100.0)	124 (100.0)
6	No plastic bag with tobacco product advertisement	229 (99.1)	141 (97.2)	134 (100.0)	137 (100.0)	108 (97.3)	99 (83.9)	98 (98.0)	115 (93.5)	100 (100.0)	124 (100.0)
7	No uniform clothing with tobacco product advertisements	230 (99.6)	145 (100.0)	134 (100.0)	137 (100.0)	111 (100.0)	118 (100.0)	100 (100.0)	123 (100.0)	100 (100.0)	124 (100.0)
8	No watches with tobacco product advertisements	231 (100.0)	145 (100.0)	134 (100.0)	137 (100.0)	111 (100.0)	118 (100.0)	100 (100.0)	123 (100.0)	100 (100.0)	124 (100.0)
9	No umbrellas with tobacco product advertisements	231 (100.0)	145 (100.0)	134 (100.0)	137 (100.0)	111 (100.0)	118 (100.0)	100 (100.0)	123 (100.0)	100 (100.0)	124 (100.0)
10	No cigarette package with inaccurate or misleading information that can attract customers	202 (87.4)	83 (57.2)	132 (98.5)	107 (78.1)	82 (73.9)	37 (31.4)	99 (99.0)	77 (62.6)	84 (84.0)	52 (41.9)
11	No price stickers that obscure health warnings on cigarette packages	231 (100.0)	129 (89.0)	133 (99.3)	137 (100.0)	110 (99.1)	84 (71.2)	100 (100.0)	123 (100.0)	99 (99.0)	117 (94.4)
12	No gifts with purchase of cigarette, special or limited time offer	230 (99.6)	145 (100.0)	134 (100.0)	137 (100.0)	111 (100.0)	114 (96.6)	100 (100.0)	123 (100.0)	100 (100.0)	122 (98.4)
13	No multi-pack discount for tobacco product	229 (99.1)	145 (100.0)	134 (100.0)	136 (99.3)	111 (100.0)	117 (99.2)	100 (100.0)	122 (99.2	100 (100.0)	124 (100.0)
14	No tobacco advertisements with culturally specific references (*such as special images*,* symbols*,* or colors*)	231 (100.0)	145 (100.0)	134 (100.0)	137 (100.0)	111 (100.0)	118 (100.0)	100 (100.0)	123 (100.0)	100 (100.0)	124 (100.0)
15	No advertisements of e-cigarettes	231 (100.0)	145 (100.0)	134 (100.0)	137 (100.0)	111 (100.0)	118 (100.0)	100 (100.0)	123 (100.0)	100 (100.0)	124 (100.0)
16	No tobacco product clearly visible from the entrance/main counter or any other part of the PoS	203 (87.9))	73 (50.3)	76 (56.7)	106 (77.4)	85 (76.6)	57 (48.3)	74 (74.0)	77 (62.6)	69 (69.0)	73 (58.9)
17	No cigarettes displayed near a cashier	222 (96.1)	108 (74.5)	102 (76.1)	106 (77.4)	100 (90.1)	64 (54.2)	83 (83.0)	114 (92.7	73 (73.0)	78 (62.9)
18	No cigarette packages displayed above the counter	230 (99.6)	127 (87.6)	118 (88.1)	115 (83.9)	109 (98.2)	73 (61.9)	91 (91.0)	121 (98.4	76 (76.0)	76 (61.3)
19	No cigarette packages displayed on shelves	206 (89.2)	82 (56.6)	89 (66.4)	108 (78.8)	95 (85.6)	60 (50.8)	75 (75.0)	78 (63.4)	73 (73.0)	75 (60.5)
20	No cigarettes displayed in any power wall	231 (100.0)	142 (97.9)	134 (100.0)	137 (100.0)	111 (100.0)	104 (88.1)	97 (97.0)	123 (100.0)	99 (99.0)	123 (99.2)
21	No cigarettes displayed near products for children	227 (98.3)	120 (82.8)	105 (78.4)	127 (92.7)	111 (100.0)	76 (64.4))	90 (90.0)	111 (90.2	77 (77.0)	87 (70.2
22	No cigarettes placed with candles	229 (99.1)	144 (99.3)	133 (99.3)	137 (100.0)	111 (100.0)	80 (67.8)	100 (100.0)	123 (100.0)	76 (76.0)	122 (98.4)
23	No cigarettes placed with attractive lights	231 (100.0)	137 (94.5)	134 (100.0)	137 (100.0)	111 (100.0)	102 (86.4)	99 (99.0)	123 (100.0)	100 (100.0)	123 (99.2)
	**Average compliance**,** n (%)**	**98.0**	**90.6**	**94.0**	**94.9**	**96.4**	**80.5**	**95.8**	**93.6**	**92.4**	**88.9**

### Outdoor compliance with tobacco advertising and promotion laws

Of 1468 PoS observed, only 52 (3.5%) had one or more outdoor tobacco advertisements, which included posters, plastic bags, and free-standing umbrellas. Using the 10 specific outdoor indicators, the average outdoor compliance was 99.6% (95% CI:99.5–99.7) (Table 4). The overall outdoor compliance for the specific indicators ranged from 98.2 to 100%. The average outdoor compliance ranged from 100% at supermarkets to 99.1% at street vendors. Assosa, Gambella, and Hawassa had 100% average compliance in outdoors.

**Table 4:** Outdoor compliance indicators of tobacco advertising and promotion laws by points-of-sale and city

Points-of-sale (*n* = 1468)	Outdoor compliance indicators, *n* (%)										
	No tobacco advertising poster	No tobacco advertising plastic bag	No tobacco advertising freestanding umbrella	No tobacco advertisements with culturally specific references	No wall paintings/ decoration with tobacco advertisement	No cigarette carton boxes	No e-cigarette advertisement	No tobacco advertising freestanding billboard	No public TV screen showing tobacco advertisement	No transit vehicles with tobacco advertisement	Average compliance
Supermarket Khat shop Minimarket Regular shop Merchandise store Permanent kiosk Food and drink wholesaler Street vendor	48 (100.0) 164 (100.0) 100 (99.0) 738 (98.1) 87 (97.8) 129 (95.6) 33 (97.1) 142 (97.9)	48 (100.0) 163 (99.4) 100 (99.0) 745 (99.1) 88 (98.9) 135 (100.0) 34 (100.0) 140 (96.6)	48 (100.0) 164 (100.0) 101 (100.0) 751 (99.9) 89 (100.0) 134 (99.3) 34 (100.0) 141 (97.2)	48 (100.0) 164 (100.0) 101 (100.0) 750 (99.7) 87 (97.8) 135 (100.0) 34 (100.0) 144 (99.3)	48 (100.0) 164 (100.0) 101 (100.0) 750 (99.7) 89 (100.0) 135 (100.0) 34 (100.0) 145 (100.0)	48 (100.0) 164 (100.0) 101 (100.0) 751 (99.9) 89 (100.0) 135 (100.0) 33 (97.1) 145 (100.0)	48 (100.0) 164 (100.0) 101 (100.0) 750 (99.7) 89 (100.0) 135 (100.0) 34 (100.0) 145 (100.0)	48 (100.0) 164 (100.0) 101 (100.0) 752 (100.0) 89 (100.0) 135 (100.0) 34 (100.0) 145 (100.0)	48 (100.0) 164 (100.0) 101 (100.0) 752 (100.0) 89 (100.0) 135 (100.0) 34 (100.0) 145 (100.0)	48 (100.0) 164 (100.0) 101 (100.0) 752 (100.0) 89 (100.0) 135 (100.0) 34 (100.0) 145 (100.0)	**100.0** **99.9** **99.8** **99.6** **99.4** **99.5** **99.4** **99.1**
**City **(*n*** = 1468)**											
Addis Ababa Adama Bahir Dar Hawassa Jigjiga Semera-Logia Dire Dawa Harar Assosa Gambella	257 (100.0) 158 (97.5) 150 (100.0) 153 (100.0) 121 (100.0) 110 (83.3) 109 (99.1) 137 (100.0) 110 (100.0) 136 (100.0)	254 (98.8) 161 (99.4) 150 (100.0) 153 (100.0) 121 (100.0) 132 (100.0) 109 (99.1) 127 (92.7) 110 (100.0) 136 (100.0)	256 (99.6) 162 (100.0) 150 (100.0) 153 (100.0) 121 (100.0) 130 (98.5) 107 (97.3) 137 (100.0) 110 (100.0) 136 (100.0)	256 (99.6) 161 (99.4) 150 (100.0) 153 (100.0) 121 (100.0) 130 (98.5) 109 (99.1) 137 (100.0) 110 (100.0) 136 (100.0)	257 (100.0) 162 (100.0) 150 (100.0) 153 (100.0) 121 (100.0) 130 (98.2) 110 (100.0) 137 (100.0) 110 (100.0) 136 (100.0)	257 (100.0) 162 (100.0) 149 (99.3) 153 (100.0) 120 (99.2) 132 (100.0) 110 (100.0) 137 (100.0) 110 (100.0) 136 (100.0)	257 (100.0) 162 (100.0) 150 (100.0) 153 (100.0) 121 (100.0) 130 (98.5) 110 (100.0) 137 (100.0) 110 (100.0) 136 (100.0)	257 (100.0) 162 (100.0) 150 (100.0) 153 (100.0) 121 (100.0) 132 (100.0) 110 (100.0) 137 (100.0) 110 (100.0) 136 (100.0)	257 (100.0) 162 (100.0) 150 (100.0) 153 (100.0) 121 (100.0) 132 (100.0) 110 (100.0) 137 (100.0) 110 (100.0) 136 (100.0)	257 (100.0) 162 (100.0) 150 (100.0) 153 (100.0) 121 (100.0) 132 (100.0) 110 (100.0) 137 (100.0) 110 (100.0) 136 (100.0)	**99.8** **99.6** **99.9** **100.0** **99.9** **97.7** **99.5** **99.3** **100.0** **100.0**
**Overall compliance**,** n (%)**	**98.2**	**99.0**	**99.6**	**99.7**	**99.9**	**99.9**	**99.9**	**100.0**	**100.0**	**100.0**	**99.6**

About 60% (95% CI:58–63) of the observed PoS were fully compliant indoors, with supermarkets showing the highest compliance (97.9%), while permanent kiosks had the lowest indoor compliance (40.0%) (Table 5). Indoor full compliance was highest in Addis Ababa (82.3%), but lowest in Semera-Logia (26.3%). Outdoor full compliance was 96.5% (95% CI:95.4–97.3), ranging from 100% at supermarkets to 91.7% at street vendors. Outdoor full compliance was 100% in Assosa, Gambella, and Hawassa, but the lowest in Semera-Logia (80.3%).

**Table 5:** Indoor and outdoor compliance status with tobacco advertising and promotion laws by points-of-sale and city

Points-of-Sale	Indoor compliance, *n* (%)				Outdoor compliance, *n* (%)*		
	Fully compliant	Good	Moderate	Poor	Fully compliant	Good	Moderate
Supermarket	47 (97.9)	1 (2.1)	0	0	48 (100.0)	0	0
Minimarket	88 (87.1)	8 (7.8)	5 (5.0)	0	99 (98.0)	2 (2.0)	0
Merchandise store	54 (60.7)	16 (18.0)	18 (20.2)	1 (1.1)	86 (96.6)	1 (1.1)	0
Food and drink wholesaler	26 (76.5)	3 (8.8)	4 (11.8	1 (2.9)	32 (94.1)	2 (5.9)	0
Regular shop	397 (52.8)	146 (19.4)	191 (25.4)	18 (2.4)	727 (96.7)	21 (2.8)	4 (0.5)
Permanent kiosk	54 (40.0)	29 (21.5)	50 (37.0)	2 (1.5)	128 (94.8)	7 (5.2)	0
Khat shop	132 (80.5)	22 (13.4)	10 (6.1)	0	163 (99.4)	1 (0.6)	0
Street vendor	-	-	-	-	133 (91.7)	11 (7.6)	1 (0.7)
**City**							
Addis Ababa	190 (82.3)	34 (14.7)	7 (3.0)	0	253 (98.4)	3 (1.2)	1 (0.4)
Adama	69 (47.6)	28 (19.3)	47 (32.4)	1 (1.7)	157 (96.9)	4 (2.5)	1 (0.6)
Hawassa	102 (74.5)	5 (3.6)	30 (21.9)	0	153 (100.0)	0	0
Bahir Dar	75 (56.0)	40 (29.2)	19 (14.2)	0	149 (99.3)	1 (0.7)	0
Jigjiga	78 (70.3)	23 (20.7)	10 (9.0)	0	120 (99.2)	1 (0.8)	0
Semera-Logia	31 (26.3)	17 (14.4)	49 (41.5)	21 (17.8)	106 (80.3)	22 (16.7)	4 (3.0)
Dire Dawa	73 (73.0)	9 (9.0)	18 (18.0)	0	105 (95.5)	4 (3.6)	1 (0.9)
Harar	69 (56.1)	33 (26.8)	21 (17.1)	0	127 (92.7)	10 (7.3)	0
Assosa	64 (64.0)	8 (8.0)	28 (28.0)	0	110 (100.0)	0	0
Gambella	47 (37.9)	28 (22.6)	49 (39.5)	0	136 (100.0)	0	0
**Total**,** n (%)**	**798 (60.3)**	**225 (17.0)**	**278 (21.0)**	**22 (1.7)**	**1416 (96.5)**	**45 (3.1)**	**7 (0.5)**

*Fully compliant: compliance with 23 indicators; Good compliance: compliance with 20-22indicators; Moderate compliance: compliance with 15–19 indicators; Poor compliance: compliance with ≤ 14 indicators

**The overall average compliance was calculated by adding up the values of ‘specific compliance indicators’ and dividing them by the total number of indicators

### Factors associated with indoor non-compliance and open display of cigarettes

Indoor non-compliance (or not fully complaint) was significantly higher in permanent kiosks (adjusted prevalence ratio (adjPR) 7.09; 95% CI: 4.31–11.68), and regular shops (adjPR 5.43; 95% CI: 3.29–8.89) than in supermarkets and minimarkets (Table 6). In addition, when compared to supermarkets and minimarkets, the PoS’s indoor open display of cigarette packages was significantly higher in merchandise/food/drink stores (adjPR 3.84; 95% CI: 2.07–7.13), and permanent kiosks (adjPR 6.73; 95% CI: 3.96–11.42). Adama (adjPR 3.06; 95% CI: 2.26–4.14) had a significantly higher indoor non-compliance when compared to Addis Ababa. Similarly, indoor open display of cigarettes was significantly higher in Adama (adjPR 4.20; 95% CI: 2.89–6.11).

**Table 6:** Multivariable Poisson regression predictors of indoor ‘open display of cigarettes’ and ‘non-compliance’ with tobacco advertising and promotion laws

Points-of-sale	Indoor open display of cigarettes		Indoor non-compliance with tobacco advertising and promotion laws	
	Adjusted Prevalence Ratio (95% CI)	*p*-value	Adjusted Prevalence Ratio (95% CI)	*p*-value
Supermarket/minimarket	Reference			
Regular shop	5.16 (3.05–8.75)	< 0.001	5.43 (3.29–8.98)	< 0.001
Permanent kiosk	6.73 (3.96–11.42)	< 0.001	7.09 (4.31–11.68)	< 0.001
Merchandise store/food and drink establishments	3.84 (2.07–7.13)	< 0.001	4.21 (2.41–7.36)	< 0.001
Khat shop	2.06 (1.11–3.83)	0.022	2.22 (1.24–3.98)	< 0.001
**City**				
Addis Ababa	Reference			
Adama	4.20 (2.89–6.11)	< 0.001	3.06 (2.26–4.14)	< 0.001
Hawassa	1.71 (1.08–2.72)	0.022	1.33 (0.90–1.97)	0.158
Bahir Dar	3.64 (2.46–5.41)	< 0.001	2.56 (1.84–3.56)	< 0.001
Jigjiga	1.57 (0.97–2.53)	0.065	1.37 (0.93–2.03)	0.115
Semera-Logia	3.25 (2.20–4.81)	< 0.001	3.19 (2.37–4.29)	< 0.001
Dire Dawa	1.52 (0.93–2.48)	0.094	1.09 (0.71–1.67)	0.705
Harar	2.95 (1.95–4.48)	< 0.001	2.38 (1.69–3.43)	< 0.001
Assosa	2.22 (1.41–3.49)	0.001	1.77 (1.21–2.60)	0.003
Gambella	3.26 (2.21–4.82)	< 0.001	3.37 (2.51–4.51)	< 0.001
Intercept	0.031 (0.017–0.031)	< 0.001	0.042 (0.024–0.074)	< 0.001

## Discussion

In this study, the average compliance with tobacco advertising and promotion laws was 92.9% for indoors and 99.6% for outdoors. About 60% and 96.5% of the PoS were fully compliant with indoor and outdoor compliance, respectively. Supermarkets and minimarkets had the highest indoor and outdoor compliance rates, and the lowest compliance rates were observed among street vendors, permanent kiosks, and regular shops. From cities, Addis Ababa and Hawassa had the highest, while Semera-Logia and Adama had the lowest indoor and outdoor compliance rates. The most common types of indoor tobacco advertising and promotion were open tobacco package displays (32.5%), having at least one tobacco product that was clearly visible from the entrance/main counter or any other part of the PoS. Tobacco advertisements using posters were more common in Semera-Logia, while those using plastic bags were more prevalent in Harar.

Studies in other LMIC countries have also found similar compliance rates for bans on tobacco advertising and promotion at the PoS. For example, a study in India observed indoor and outdoor compliance with cigarette advertisements at PoS of 42% and 91%, respectively [25]. In another study in Panama, indoor tobacco product advertising was observed only in 5.4% of PoS [31], which was comparable to the results of our study (5%). This study found that open displays of tobacco packages at the PoS was the main indoor tobacco advertising and promotion mechanism. Open cigarette displays in the PoS, particularly using shelves, were clearly and easily noticeable in the current study. The findings of this study are lower compared to the findings in Indonesia [4] and China [32], where the prevalence of tobacco smoking is very high. In Indonesia, a country that has not ratified the WHO FCTC, cigarettes were openly displayed by the majority of the retailers (98.9%) [4]. In China, almost all cigarette stores (97%) and e-cigarette retailers (86.7%) displayed tobacco products at the PoS, with the majority displayed in a way that was visible from the main entrance of the shop [32]. In Amsterdam, the Netherlands [33], 91.5% of the 82 studied PoS had indoor open displays of tobacco products. Overall, this study reported a lower percentage of PoS with open cigarette package displays compared to countries with high tobacco consumption or those not yet ratified with the WHO FCTC, but the percentage is still unacceptable, indicating the need for improved TAPS law enforcement and increased community awareness about these laws.

The existing evidence suggests a positive association between exposure to tobacco advertising at PoS and smoking [9]. Studies have shown that partial and full implementation of open tobacco display bans can significantly reduce smoking susceptibility among adolescents [18]. According to a recent study of tobacco display legislation in Scotland [34], the implementation of both partial and comprehensive PoS tobacco display bans was associated with reduced perceived accessibility of tobacco products and a more negative attitude toward tobacco. Following the implementation of tobacco display bans at PoS in Finland [35], a significant decrease in adolescent exposure to tobacco products was reported. The removal of PoS tobacco displays in Norway [36] resulted in 97% compliance for cigarettes.

The open display of tobacco packages was prevalent in the current study, suggesting that this is one main mechanism that tobacco companies are using to market their products in Ethiopia. This could be attributed to the weak enforcement of tobacco control laws, particularly in cities other than Addis Ababa, coupled with low awareness about tobacco control laws among PoS owners. Addis Ababa FMHACA conducted awareness creation activities about TAPS laws for shop owners, including those of minimarkets and supermarkets, months before the data collection period for this study. The 2019 Tobacco Proclamation [17] of Ethiopia bans the open display of tobacco products, as well as any other tobacco advertising and promotion at the PoS, and directs that tobacco products be placed or stored under or behind the counter, where customers cannot directly view or grasp the product.

Studies have shown that tobacco advertising and promotion compliance at PoS varies across and within nations, which might mean that the enforcement of tobacco control policies need to be adjusted to local circumstances. For example, we found that compliance with indoor and outdoor tobacco advertising and promotion laws varied significantly across PoS and cities, with compliance being highest in supermarkets, minimarkets, and among the PoS in Addis Ababa and Hawassa, but lowest in regular shops and permanent kiosks, as well as in the PoS in Adama, Gambella, and Semera-Logia. The differences between the cities could be explained by the socio-cultural differences, smoking prevalence differences, and varying levels of tobacco control enforcement. Most people buy cigarettes (especially single sticks) from regular shops, khat shops or kiosks, with cigarettes not being a common commodity in supermarkets or minimarkets.

Higher compliance at supermarkets and minimarkets may also be due to increased information about the recent stringent tobacco advertising and promotion laws, which include a potential store closure and accompanying penalties, notably observed in Addis Ababa. Variations in compliance with tobacco advertising and promotion laws by type of PoS were also reported in Indonesia [37], with larger shops having the highest compliance rates. Therefore, the types of PoS and geographic areas should be taken into account in order to strengthen the enforcement of tobacco advertising and promotion laws. It should be noted, however, that high compliance rate does not necessarily indicate strong enforcement of tobacco control polices. In supermarkets, for example, commodities are normally sold in packs rather than as single items, and this is also true for cigarettes.

### Strengths

To the best of our knowledge, this is the first comprehensive study that evaluated compliance with tobacco advertising and promotion laws across a diverse settings and various retailers of tobacco products in Ethiopia. The use of a detailed composite indoor indicator encompassing 23 specific compliance indicators offers a strong measure of compliance to tobacco control laws. The inclusion of both indoor and outdoor compliance provides a holistic view of the regulatory landscape. Additionally, the study’s large sample size and the variety of PoS types analyzed enhance the reliability and generalizability of the findings within the urban Ethiopian context.

### Limitations

Despite the comprehensive nature of this study, there are several limitations to consider. The data represents a snapshot in time and may not capture seasonal variations or changes in compliance over time. While the study covered multiple cities, it may not fully represent rural areas or smaller towns where tobacco advertising practices could differ significantly. Additionally, the findings may not be directly applicable to other countries or regions with different regulatory environments, cultural norms, and market structures, highlighting the importance of context-specific tobacco control strategies.

### Conclusions and recommendations

This study underscores the importance of robust enforcement and tailored interventions to improve compliance with tobacco advertising and promotion laws in Ethiopia. Overall, supermarkets, minimarkets, and PoS in Addis Ababa had better indoor and outdoor compliance rates, while compliance rates were lower among permanent kiosks, regular stores, PoS in Semera-Logia and Adama. The high overall compliance rates are encouraging, but the significant disparities between different PoS types and cities highlight specific areas that require targeted interventions. Policymakers and enforcement agencies must prioritize strengthening compliance mechanisms, particularly in low-compliance areas and among specific PoS types. Enhanced public awareness and education campaigns, training programs for PoS owners and retailers, combined with stricter penalties and regular monitoring, can drive better compliance to tobacco control laws. Continued research and evaluation are essential to refine these strategies and ensure sustained progress in tobacco control efforts.

### Electronic supplementary material

Below is the link to the electronic supplementary material.

Supplementary Material 1

Supplementary Material 2

Supplementary Material 3

## Data Availability

Data is provided within the manuscript or supplementary information files. In addition, the full datasets used and analyzed in the current study are available from the corresponding author upon reasonable request.

## Abbreviations

AdjPR: Adjusted Prevalence Ratio
CI: Confidence Interval
DSAs: Designated smoking areas
EFDA: Ethiopian Food and Drug Authority
EPHA: Ethiopian Public Health Association
FCTC: Framework Convention on Tobacco Control
LMICs: Low- and middle-income countries
ODK: Open Data Kit
PoS: Points-of-salet
TAPS: Tobacco advertising, promotion, and sponsorship
VIF: Variance Inflation Factor
WHO: World Health Organization
