# Supplementary figures and images for: Compliance with tobacco advertising and promotion laws at points-of-sale in Ethiopia: an observational study in 10 cities
Source: BMC Public Health. 2024 Jul 22;24:1952. doi: 10.1186/s12889-024-19478-7 (PMC11265118; doi:10.1186/s12889-024-19478-7)

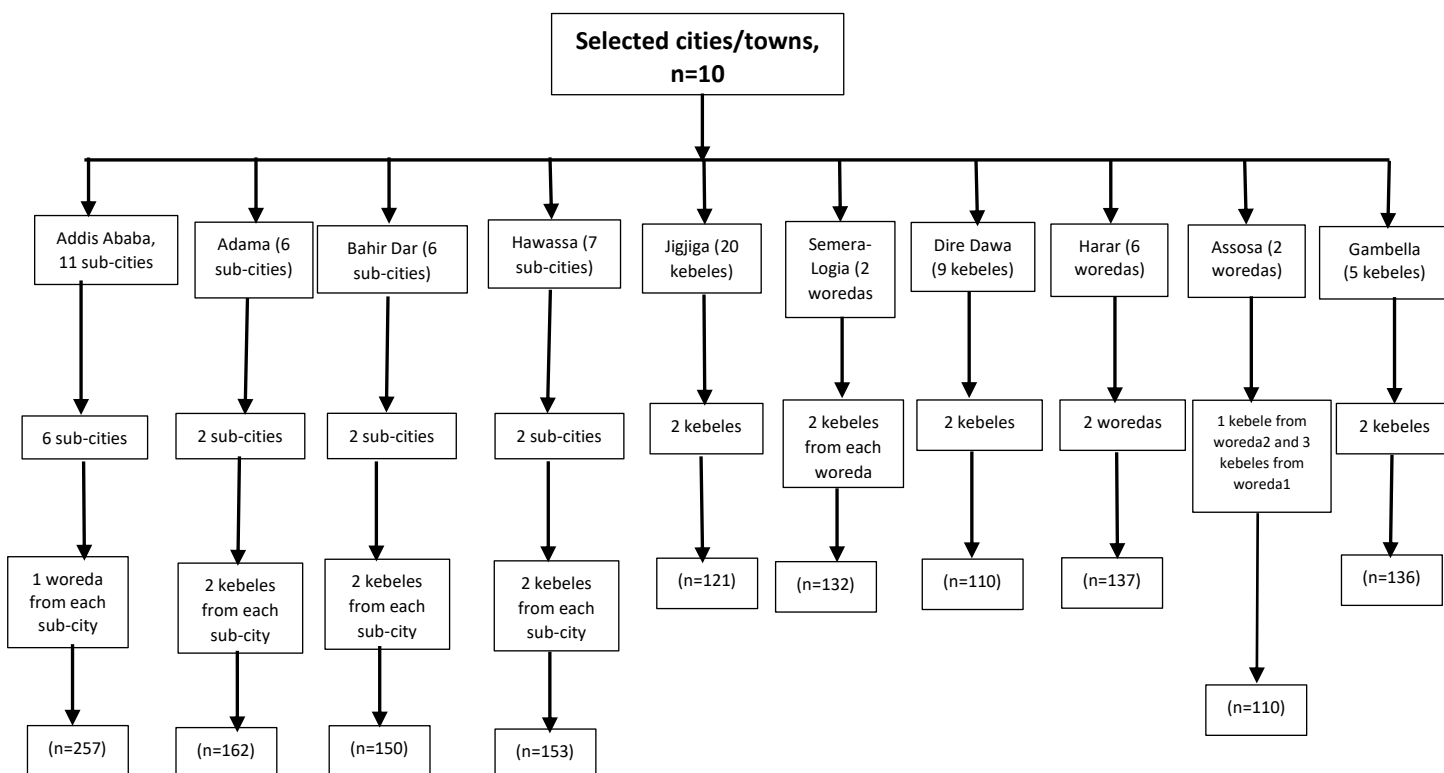

**S1. Multistage schematic sampling technique, Ethiopia, December 2022**

Supplement: Supplementary file 1 — Supplementary Material 1 [file 12889_2024_19478_MOESM1_ESM.pdf]
