# Supplementary material for: Compliance with tobacco advertising and promotion laws at points-of-sale in Ethiopia: an observational study in 10 cities
Source: BMC Public Health. 2024 Jul 22;24:1952. doi: 10.1186/s12889-024-19478-7 (PMC11265118; doi:10.1186/s12889-024-19478-7)
Supplement: Supplementary file 2 — Supplementary Material 2 [file 12889_2024_19478_MOESM2_ESM.pdf]

S2. Observation Checklists for Points of Sale

Observation form for tobacco advertising and promotion activities

Section I: Point of Sale Identifier

Variable Name	QUESTIONS	Response/response categories	Skip
POSID	Unique ID for the point of sale (POS)		
POS101	Administrative Region	1. Addis Ababa<br>2. Oromia<br>3. Amhara<br>4. Sidama<br>5. Somali<br>6. Afar<br>7. Dire Dawa<br>8. Harari<br>9. Benishangul Gumuze<br>10. Gambela	
POS102	City/Town	1. Addis Ababa<br>2. Adama<br>3. Bahir Dar<br>4. Hawassa<br>5. Jigiga<br>6. Semera -Logia<br>7. Dire Dawa<br>8. Harar<br>9. Assosa<br>10. Gambela	
POS103	Zone/sub-city		
POS104	Woreda		
POS105	Kebele		
POS106	Date of the visit (DD/MM/YY)	|__|__| |__|__| |__|__| **dd mm year**	
POS107	Type of the point of sale	1. Supermarket<br>2. Khat shop<br>3. Merchandise Store<br>4. Mini market<br>5. Regular shop<br>6. Permanent kiosk<br>7. Street vendor<br>8. Food and drink wholesalers<br>96. Other, please specify__________	

Section II: Outdoor TAPS activities

**Variable Name**	**QUESTIONS**	**Response (**For each item, please select "0" if you don't observe it today.<br>Please enter "1" if you observe/ available today.		**Skip**
POS201	Is there an outdoor advertisement?	0.No	1.Yes	If "No" go to POS208
POS202	If yes to question POS201, observe each of the following			
a.	Poster	0.No	1.Yes	
b.	Wall painting/decoration	0.No	1.Yes	
c.	Freestanding billboard	0.No	1.Yes	
d.	Freestanding umbrella	0.No	1.Yes	
e.	Public TV screen	0.No	1.Yes	
f.	Transit vehicles	0.No	1.Yes	
x.	Other ________________________	0.No	1.Yes	
POS203	At least one advertisement is clearly visible from a point of regular pedestrian or vehicle traffic?	0.No	1.Yes	
POS204	Presence of health warnings on ads that are comprised of combined images and full-color pictures.<br>[The health warnings and messages shall be displayed no less 70% of the front and back side of each principal display area of its packaging and labeling].	0.No	1.Yes	
POS205	Does the advertisement use culturally specific references *(such as special images, symbols, or colors, etc.)* in at least one of the tobacco products*?*	0.No	1.Yes	
POS206	Do you observe **smokeless tobacco or** flavored tobacco products in at least one of the advertisements?	0.No	1.Yes	
POS207	Do you observe e-cigarettes in at least one of the advertisements?	0.No	1.Yes	
POS208	Presence of any person smoking tobacco (within 10meter radius of the shop)	0.No	1. Yes	
POS209	Were there any schools, health institutions or youth centers within 100 meters radius of the premises of the POS?	0.No	1.Yes	

POS 210	Do you observe cigarette butts in the outdoor space?			
POS211	Any additional information about the outdoor observation			

SECTION III: Indoor Observation

**Variable Name**	**QUESTIONS**	**Response (**For each item, please select "0" if you don't observe it today. Please enter "1" if you observe/ available today.		**Skips**
	**Advertisement**			
POS301	Is any one of the advertisements on tobacco product or tobacco industry (containing logo mark or symbol of tobacco) clearly visible from the entrance or main counter/any other part of the shop?	0.No	1.Yes	If 'no' go to POS 303
POS302	If yes to POS301, which one **is** visible?			
a	Posters	0.No	1.Yes	
b	Stickers	0.No	1.Yes	
c	Video screens	0.No	1.Yes	
d	Furniture/objects	0.No	1.Yes	
e	Plastic bag			
F	Uniforms	0.No	1.Yes	
G	Watch	0.No	1.Yes	
g	Umbrella	0.No	1.Yes	
h	Other specify.			
POS 303	Do you observe a cigarette package?	0.No	1. Yes	If 'no' go to POS 306
POS304	Please observe the tobacco package that attracts consumers. Any inaccurate information, misleading statement, trademark, figure, color, or other sign of any kind that directly or ' in directly creates or likely to create the false impression that a particular tobacco product is ' less harmful than others, including the term "low tar, "light", "ultra-light", or mild, "extra", and "ultra " or any kind of flavor.			
a	Color	0.No	1.Yes	
b	Message	0.No	1.Yes	
c	Picture/figure	0.No	1.Yes	
d	Other, please specify_______________			

POS305	Price stickers that obscure health warnings on the package of tobacco products.	0. No	1. Yes	
POS306	Presence of gift with purchase, special or limited time offer	0.No	1.Yes	
POS307	Presence of multi-pack discounts	0.No	1.Yes	
POS308	Use of culturally specific references on ad *(such as special images, symbols, or colors, etc.)*	0.No	1.Yes	
POS309	Advertisement of smokeless tobacco or flavored tobacco products.	0.No	1.Yes	
POS310	Advertisement of e-cigarettes	0.No	1.Yes	
	**Product Placement (Displays)**			
POS311	Observe the tobacco product placements (containing the logo, symbol, brand name, and/or color of tobacco products).			
a-	Can the tobacco products be directly seen?	0.No	1.Yes	If "no" skip to POS 316
b-	If yes to the above question, which ones?	1- Nyala Premium<br>2- Nyala<br>3- Gisilla<br>4- Delight<br>5- Elleni<br>6- Marlboro gold<br>7- Marlboro<br>8- Rothmans royals<br>9- Rothmans<br>10- Winston<br>11- Oris<br>12- Green apple<br>13- Strawberry<br>14- Sports man<br>96. Other ____________		
c-	Can a customer directly grasp the tobacco product?	0.No	1.Yes	If "no" skip to e
d-	If yes to the above question, which ones?	1- Nyala Premium<br>2- Nyala<br>3- Gisilla<br>4- Delight<br>5- Elleni<br>6- Marlboro gold<br>7- Marlboro<br>8- Rothmans royals<br>9- Rothmans<br>10- Winston		

		11- Oris<br>12- Green apple<br>13- Strawberry<br>14- Sports man<br>96. Other		
e-	Cigarettes displayed near a cashier	0.No	1.Yes	
f-	Cigarettes displayed on the countertop	0.No	1.Yes	
g-	Cigarettes displayed on shelves	0.No	1.Yes	
h-	The height of the display >1m	0.No	1.Yes	
POS312	Cigarette displayed in any power wall?	0.No	1.Yes	If "No" skip to POS 316
POS 313	If yes to question POS312, built display cabinet for tobacco products by specific cigarette company/brand name	0.No	1.Yes	
POS314	Built display cabinet for tobacco products only	0.No	1.Yes	
POS315	Built display cabinet for tobacco products and non-tobacco product	0.No	1.Yes	
POS316	Cigarettes placed near products for children	0. No	1.Yes	
POS 317	Cigarettes placed with candles	0.No	1.Yes	
POS318	Cigarettes placed with attractive lights	0.No	1.Yes	
	**Warning Signage**			
POS319	Presence of clear and prominent posts regarding the prohibition of tobacco smoking and use along with its corresponding **"no-smoking sign".**	0.No	1.Yes	
POS320	Presence of post/notice regarding **"no tobacco sells for age less than 21 years"**	0.No	1.Yes	
	**Presence of tobacco consumption**			
POS 321	**Do you observe anyone smoking in the indoor space of the retail point?**	0. No	1. Yes	
POS 322	**Do you observe a cigarette butt in the indoor space?**			
POS 323	**Do you observe a lighter?**	0.No	1. Yes	
POS324	Do you observe a single stick sale of cigarettes in the POS?	0.No	1.Yes	
POS 325	Any additional information about the indoor observation.			
