# Supplementary material for: Compliance with tobacco advertising and promotion laws at points-of-sale in Ethiopia: an observational study in 10 cities
Source: BMC Public Health. 2024 Jul 22;24:1952. doi: 10.1186/s12889-024-19478-7 (PMC11265118; doi:10.1186/s12889-024-19478-7)
Supplement: Supplementary file 3 — Supplementary Material 3 [file 12889_2024_19478_MOESM3_ESM.pdf]

S3. Operational definitions

Food and drink wholesaler: means any person who sells food products and beverages to retailers or governmental and non-governmental organizations or cooperatives by wholesale after having bought such goods from producers or importers.

Indoor: means any place within a retail establishment that has a fully enclosed and secure structure with an entrance. This definition doesn't include street or mobile vendors.

Khat shop: is an establishment where khat leaves (Catha edulis) are sold. It is typically a permanent structure, often found in buildings or housing complexes, catering to customers purchasing khat for personal consumption. These shops often have counters and shelves for displaying and selling khat, as well as other products such as cigarettes, alcoholic and non-alcoholic beverages, snacks, and other goods to complement the sale of khat.

Merchandise store: a store that is big and sells a variety of things such as food products and beverages, and household supplies including clothes.

Minimarket: refers to a store that sells food and sometimes other goods but is not as big as a supermarket.

Outdoor: means any place outside of any retail establishment that is not 'indoors', including any verandah, exterior wall or a window facing outward in any such establishment. This definition also includes street or mobile venders.

Permanent kiosk: refers to a small, permanent, stand-alone venue/location used for marketing purposes.

Regular shop: a small retail store in a building or part of a building where certain goods including food products and drink items are sold or purchased.

Street vendor: a person who offers goods or services for sale to the public without having a permanently built structure but with a temporary static structure with an open front on the street.

Supermarket*:* a large type of retail store that generally sells a range of household items, including food and beverages, sanitary materials, and cosmetics, and is properly placed and arranged in specific departments.

Tobacco product: any product entirely or partly made of the tobacco leaf as a raw material which is manufactured to be used for smoking, chewing, sucking, or snuffing.
